# Carica Papaya Reduces High Fat Diet and Streptozotocin-Induced Development of Inflammation in Adipocyte via IL-1β/IL-6/TNF-α Mediated Signaling Mechanisms in Type-2 Diabetic Rats

**DOI:** 10.3390/cimb45020056

**Published:** 2023-01-18

**Authors:** Jeane Rebecca Roy, Coimbatore Sadagopan Janaki, Selvaraj Jayaraman, Vijayalakshmi Periyasamy, Thotakura Balaji, Madhavan Vijayamalathi, Vishnu Priya Veeraraghavan, Kalaiselvi Krishnamoorthy, Monisha Prasad

**Affiliations:** 1 Department of Anatomy, Bhaarath Medical College and Hospital, Bharath Institute of Higher Education and Research (BIHER), Chennai 600 073, Tamil Nadu, India; 2 Centre of Molecular Medicine and Diagnostics (COMManD), Department of Biochemistry, Saveetha Dental College & Hospitals, Saveetha Institute of Medical & Technical Sciences, Saveetha University, Chennai 600 077, Tamil Nadu, India; 3 Department of Biotechnology and Bioinformatics, Holy Cross College, Trichy 620 002, Tamil Nadu, India; 4 Department of Anatomy, Chettinad Hospital and Research Institute, Chettinad Academy of Research and Education, Chennai 603 103, Tamil Nadu, India; 5 Department of Physiology, Bhaarath Medical College and Hospital, Bharath Institute of Higher Education and Research (BIHER), Chennai 600 073, Tamil Nadu, India

**Keywords:** Carica papaya, adipose tissue, inflammatory signaling, mTOR, TNF-α, IL-1β, IL-6, IKKβ, molecular dynamics, metabolic disorder

## Abstract

The prevalence of obesity in contemporary society has brought attention to how serious it is all around the world. Obesity, a proinflammatory condition defined by hypertrophied adipocytes and immune cells that reside in adipose tissue, is characterized by elevated circulating levels of proinflammatory cytokines. The pro-inflammatory mediators trigger a number of inflammatory pathways and affect the phosphorylation of a number of insulin-signaling pathways in peripheral tissues. In this work, we pointed the outcome of the leaves of Carica papaya (C. papaya) on the inflammatory molecules by in vivo and in silico analysis in order to prove its mechanisms of action. Adipocytokines, antioxidant enzymes, gene and protein expression of pro-inflammatory signaling molecules (mTOR, TNF-α, IL-1β, IL-6 and IKKβ) by q-RT-PCR and immunohistochemistry, as well as histopathological analysis, in adipose tissues were carried out. C. papaya reinstated the levels of adipocytokines, antioxidant enzymes and mRNA levels of mTOR, TNF-α, IL-1β, IL-6 and IKKβ in the adipose tissues of type 2 diabetic rats. Molecular docking and dynamics simulation studies revealed that caffeic acid, transferulic acid and quercetin had the top hit rates against IKKβ, TNF-α, IL-6, IL-1β, and mTOR. This study concludes that C. papaya put back the altered effects in fatty tissue of type 2 diabetic rats by restoring the adipocytokines and the gene expression.

## 1. Introduction

Obesity has become more common in modern society, which has highlighted how serious it is around the world. Both developed and developing nations view obesity as a health catastrophe. An excessive deposition of adipose tissue that negatively impacts both overall health and well-being is what constitutes obesity and being overweight [1,2]. This is in turn linked to many devastating conditions and among them is type 2 diabetes mellitus (T2DM), which draws attention globally. It is expected globally to experience the steepest rate of growth for diabetes in the next coming years, with a predicted 50% increase by 2030 [3].

The risk for T2DM can be increased by obesity through induction of insulin resistance. Increased circulating levels of proinflammatory cytokines are a hallmark of obesity, which is a proinflammatory state characterized by hypertrophied immune cells and adipocytes that dwell in adipose tissue [4,5]. A focal point in the aetiology of type 2 diabetes and insulin resistance in both human and rodent animal models is thought to be the obesity-associated condition of chronic low-grade systemic inflammation known as “metabolic inflammation” [5,6].

Inflammation, a physiological condition is defined by an increase in white blood cell count or pro-inflammatory cytokine levels in the blood or tissue. Normally, inflammation is necessary for wound healing, immunity against infections, tissue repair, and organ remodeling. An excessive inflammatory response typically has a number of negative side effects, including organ and tissue damage [7]. Increased macrophage infiltration and pro-inflammatory cytokine production in adipose tissue and the liver signal the beginning of the inflammation linked to obesity. Systemic inflammation is brought on by the pro-inflammatory cytokines that enter the bloodstream [7,8]. A set of hormones and signaling molecules known as adipokines are released by adipose tissue and have biological actions that can be systemic, autocrine or paracrine. They have an impact on a variety of physiological processes, including the generation of energy, metabolism of glucose and the immune system [9].

Adipocytes become abnormal due to glucolipotoxicity and the production of inflammation in the cells. When adipocytes are injured, glucose consumption decreases and free fatty acids (FFA) levels abnormally rise, increasing metabolic stress in adipocytes and ultimately liberated excessive pro-inflammatory mediators and adipocytokines [10,11,12]. Their aberrant release triggers a number of inflammatory pathways and affects the phosphorylation of a number of insulin-signaling pathways in peripheral tissues [10,12]. Inhibitor of nuclear factor kappa-B kinase subunit beta (IKKβ) triggers nuclear factor kappa B (NF-κB) and over phosphorylation of IKKβ facilitates NF-κB in triggering various pro-inflammatory mediators such as interleukin-6 (IL-6), tumor necrosis factor alpha (TNF-α) and interleukin 1 beta (IL-1β) [13]. Obesity is associated with an increase in the secretion of adipocytokines, such as TNF-α, leptin, resistin, IL-1β or IL-6, by immune cells as well as adipocytes, which leads to the progress of insulin resistance through a number of processes, including the activation of Ser/Thr kinases. Pro-inflammatory cytokines like leptin, IL-6 and TNF-α are noted to be formed in adipose tissues during pathogenesis of metabolic syndrome, and the anti-inflammatory cytokine, adiponectin has the power to counteract their harmful sequels. In obese conditions, the levels of adiponectin are decreased significantly and thus fail to counteract the levels IL-6, TNF-α, leptin and resistin [14,15]. Insulin signal transduction is inhibited by IL-1β, which reduces insulin sensitivity in adipose tissue. In human adipocytes, insulin-signaling and action are improved by inhibiting IL-1β activity, receptor binding or production [16]. mTOR limits the synthesis of pro-inflammatory cytokines through the inhibition of nuclear factor-κB (NFκB) activity, Adipokines like leptin activates Akt/mTOR pathway but in obese and inflammation conditions, over secretion of leptin in turn over-activates the mTOR pathway that paves the way to insulin resistance [17].

Numerous anti-inflammatory medications, such as metformin, thiazolidinediones, etc., are frequently used to treat inflammation and insulin resistance in T2DM [18]. Due to their negative side effects, the long use administration is greatly confined. To overcome this deplorable situation, researchers and physicians are now considering employing natural medications to treat insulin resistance and the inflammatory environment. Numerous plants have been reported to have anti-inflammatory effects. Carica papaya (C. papaya) is prominently featured on the list among them [19,20]. Since ancient times, several C. papaya parts have been employed for therapeutic purposes. Although many studies have concentrated on the anti-inflammatory properties of C. papaya’s leaves, fruits and seeds, the molecular mechanisms are still unclear [20,21]. In order to demonstrate the bioactive components of C. papaya’s defense against the development of pro-inflammation, we focused on the effects of the C. papaya leaves on inflammatory signaling molecules like mTOR, TNF-α, IL-1β, IL-6 and IKKβ through in vivo, molecular docking and molecular dynamics.

## 2. Materials and Methods

### 2.1. Chemicals

All of the chemicals, primers, reagents and ELISA kits utilized during this investigation were purchased from Eurofins Genomics India Pvt Ltd., Bangalore, India; Sisco Research Laboratories (Mumbai, India); Sigma Aldrich (St. Louis, MO, USA); Ray Biotech Suite (St. Louis, MO, USA); MP Biomedicals (Santa Ana, CA, USA); Abbkine Scientific Co., Ltd. (Santa Barbara, CA, USA).

### 2.2. Collection and Authentication of Plant Material

The C. papaya leaves were gathered in Kerala and was authenticated by the National Institute of Siddha, Chennai. Certificate No: NISMB4392020.

### 2.3. Preparation of Ethanolic Extract of C. papaya

The leaves of C. papaya were shade-dried and pulverized. About 1 kg of the pulverized material was packed in the Soxhlet apparatus and extracted with 95% ethanol. The extraction was carried out for 72 h at about 50 °C. Later on, the extract was filtered, and the filtrate was concentrated to a dry mass by simple distillation. The yield was found to be 6% w/v. and the extract was stored in desiccators at room temperature until analysis.

### 2.4. Animals

In accordance with the Institutional Animal Ethical Committee approval, male Wistar albino rats of 150–180 days old were housed under conventional environmental settings of ambient temperature and humidity at the Central Animal House of Saveetha Dental College and Hospital in Chennai, Tamil Nadu, they were given regular pellet food and unlimited access to water (IAEC No: BRULAC/SDCH/SIMATS/IAEC/08-2021/071 dated 21 August 2021).

### 2.5. Induction of T2DM

For four weeks, the rats were fed a high-fat diet (HFD) which consisted of 30% coconut oil, 3% cholesterol and 66 percent conventional rat feed. A low dose of streptozotocin (STZ), 35 mg/kg was intraperitoneally administered to the animals [22]. Rodents with fasting blood glucose levels (>120 mg/dL) were intended for the study after the subsequent two days of STZ administration. During the investigation, T2DM rats were given access to HFD and sucrose water.

### 2.6. Experimental Design

Five groups of eight rats each were taken using random selection. Control rats were served as Group 1; T2DM-induced rats served as Group 2; T2DM rats treated with C. papaya ethanolic leaf extract (600 mg/kg bwt for 45 days) served as Group 3; T2DM rats treated with metformin (50 mg/kg bwt for 45 days) served as Group 4 and control rats treated with C. papaya ethanolic leaf extract (600 mg/kg bwt for 45 days) served as Group 5. The administration of C. papaya and metformin were given orally to the rats.

Thiopental (40 mg/kg body weight) was used to sedate the rats on the final day of the experiment. Blood was collected via cardiac puncture, sera were isolated and the blood was kept at −80 °C. By injecting 20 mL of isotonic NaCl solution via the left ventricle, the blood was withdrawn, and the adipose tissue was instantly removed and anatomized for the parameters listed below.

### 2.7. Serum Adiponectin, Leptin and Resistin

According to the manufacturer’s instructions, serum levels of leptin, adiponectin and resistin were measured using a rat insulin ELISA kit from Ray Biotech (3607 Parkway Lane, IL, USA). <10.0% for the intra-assay coefficient of variance and <12.0% for the inter-assay coefficient of variation. Leptin levels were indicated in pg/mL, whilst adiponectin and resistin results were expressed in ng/mL.

### 2.8. Oxidative Stress Markers

Analysis of oxidative stress markers like lipid peroxidation (LPO) and hydrogen peroxide (H_2_O_2_) in the adipose tissue of the rats were observed with the kit of Abbkine Scientific Co., Ltd., CA, USA.

### 2.9. Enzymatic Antioxidants

Analysis of enzymatic markers of antioxidants like superoxide dismutase (SOD), glutathione peroxidase (GPx), reduced glutathione (GSH) and catalase (CAT) and the adipose tissue of the rats were observed with the kit of Abbkine Scientific Co., Ltd., CA, USA.

### 2.10. Total RNA, cDNA Synthesis and Real-Time PCR

TRIR kit was used to isolate total RNA from the fatty tissues of rats belonging to the five groups. Eurogentec (Seraing, Belgium) provided the reverse transcriptase kit. 2 µg of total RNA were used to produce the cDNA. Table 1 lists the primers’ sequences for this study. β-actin is chosen as the reference gene. The following reaction conditions were used to amplify the genes in a real-time PCR system (Stratagene MX 3000P, La Jolla, CA, USA): 40 cycles of 95 °C for 30 s, 59–60 °C for 30 s and 72 °C for 30 s. From the examination of the melt and amplification curves, relative quantification was derived.

### 2.11. Histopathology

The histopathology of adipose tissue in 10% neutral buffered formalin preserved in paraffin, sectioned and stained with hematoxylin and eosin [28]. About 0.5–1 microns of sections were procured by microtome and images at a magnification of ×100 were taken.

### 2.12. Immunohistochemical Analysis

About 4 µm of adipose tissues from the research animals were deparaffinized and rehydrated with xylene and ethanol, respectively, at gradually lessened percentages. The tissues were put together with sodium citrate buffer (1 M, pH 6.0–6.2) and heated for 3 cycles of five minutes each, set apart by one minute. The slides were then prepared with 1 M PBS for five minutes to prevent endogenous. Peroxidase activity was carried out in a darkened, humidity chamber for 10 min using 30% H_2_O_2_, then for 5 min using 1 M PBS as a wash. The slides were blocked with 2% bovine serum albumin and then rinsed twice with PBS for five minutes each (BSA). mTOR, TNF-α, IL-1β, IL-6 and IKKβ were used as the primary antibodies, and before processing the sections, they were diluted 1:100.

### 2.13. Statistical Analysis

The data were analyzed to check the significance of individual variance within the control and treated groups using one-way analysis of variance (ANOVA) and Duncan’s multiple range test by means of computer-based software (Graph pad prism version 5). Significance was considered at the levels of *p* < 0.05.

### 2.14. Molecular Docking

#### 2.14.1. Preparation of Protein

Three-dimensional crystal structure includes IL-1 beta (PDB ID: 9ILB), IL-6 (PDB ID:4NI9) IKKβ (PDB ID: 3BRV) human mTOR (PDB id: 4DRI), TNF (PDB id: 1TNF) have been retrieved in PDB format from RCBS Protein Data Bank. Using Discovery studio 2020, the proteins were free of water and heteroatoms.

#### 2.14.2. Ligand Preparation

Based on the literature survey on the compounds isolated from C. papaya, eight compounds were selected for docking studies (Table 2). The 2D structures of these compounds were downloaded in SDF (Spatial Data File) format from the PubChem database and then it was converted into the 3D PDB format using online smiles translator.

#### 2.14.3. Molecular Docking

The Discovery Studio module, Ligand Fit was used to carry out molecular docking analyses. The three phases of the Ligand Fit methodology are: (i) docking, which comprises trying to dock a ligand into a user-defined binding site; (ii) in place ligand reductions, and (iii) scoring, which is generating a number of grading functions for each ligand pose. The ligands were docked into the receptor’s active region after the protein was prepared for docking. The search for active sites was conducted using a flood filling method. The area of the receptor that is within 12 of the geometric centroids of the ligand was designated as the active site. The best poses were chosen from an over-all of 10 poses that were created throughout docking are based on dock score values acquired after energy reduction in the active site. 

#### 2.14.4. Investigation of Potential Compounds’ Molecular Dynamics and Simulation and Receptor Complex

The GROMACS simulation tool was used to run all-atom MD simulations for 100 ns at 300 K [29]. The receptor coordinate file was searched for additional and missing residues using the discovery studio platform. The free and docked complexes were solvated in a cubic box with a dimension of 1.0 nm using the SPC water model and neutralised them with sodium ions. Ligand topology file was created using the PRODRG server [30]. In order to minimise energy consumption, the steepest descent method was used at 1500 ps. Initially set to 0 K, the system temperature increased to 300 K during the equilibration phase. Then, with a constant pressure of 1 bar, an equilibration time of 100 ps with constant volume was accomplished under episodic boundary conditions. Utilizing Xmgrace, graphs were produced using the results of the MD simulation [31]. MM-PBSA computations were done on docked complexes. The final MD run for each system lasted 100 ns, and the generated trajectories were evaluated using the specialised modules of GROMACS. The solvent accessible surface area (SASA), radius of gyration (Rg), Root Mean Square Deviation, and Root Mean Square Fluctuation parameters were computed using the GROMACS simulation software to examine the stability of the simulation. To determine the stability of the protein-ligand complex, we counted the hydrogen bonds and measured the distance between interacting amino acids and the ligand. For each individually docked complex with a conformational shift lasting up to 100 ns, several energies were examined overall. It was generated using the GROMACS 5.0.7 program’s g mmpbsa package and the Molecular Mechanics Poisson-Boltzmann Surface Area method.

#### 2.14.5. Binding Energy Calculations

MM-PBSA is utilized for the investigation of biomolecular interactions and computational drug design. Utilizing g mmpbsa, the MM-PBSA binding energy of selected compounds was determined. 

## 3. Results

### 3.1. Effect of C. papaya on Serum Adiponectin, Leptin and Resistin

**Figure 1:**
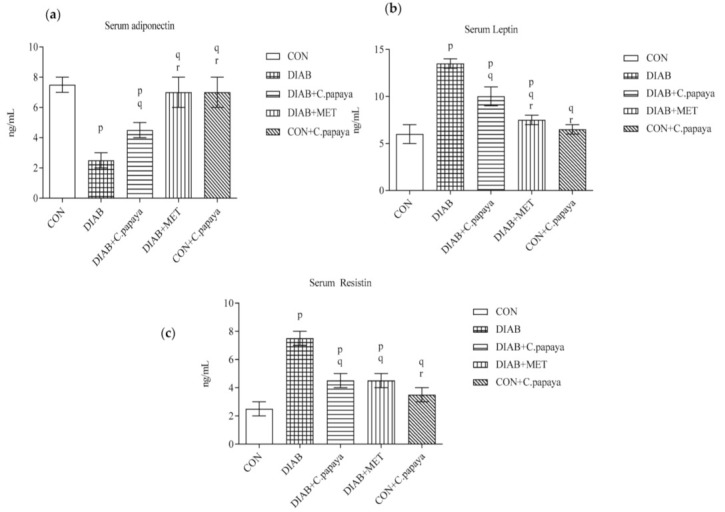
(**a**–**c**) Outcome of C. papaya on serum adiponectin, leptin and resistin levels in control and treatment rats. Each bar depicts the mean ± SEM of eight rats, with *p* < 0.05 designating statistically significant differences between the groups as follows: p—control; q—diabetes; r—T2DM rats + C. papaya.

Adiponectin, leptin and resistin belongs to the group of apparent serum markers of metabolic syndrome. On that account, their levels were investigated in the serum of control and experimental rats. Figure 1a–c showed a substantial upsurge (*p* < 0.05) in leptin and resistin concentration in addition with a significant decline in the adiponectin level in T2DM group 2 rats. The medicament with C. papaya reinstated the modified levels of adipokines in T2DM rats, such as that of the metformin administered group 4.

### 3.2. Result of C. papaya on Oxidative Stress Markers

**Figure 2:**
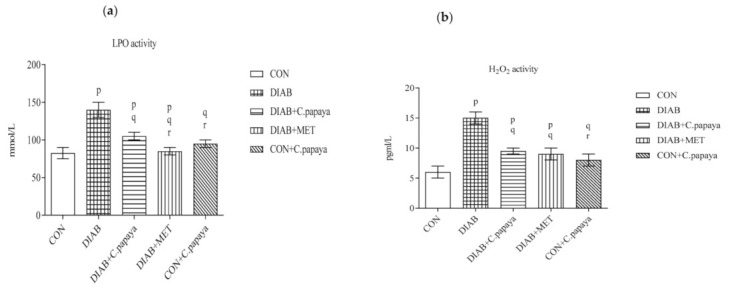
(**a**,**b**) Outcome of C. papaya on the oxidative stress marker levels in the control and treated rats. Each bar depicts the mean ± SEM of eight rats, with *p* < 0.05 designating statistically significant differences between the groups as follows: p—control; q—diabetes; r—T2DM rats + C. papaya.

The levels of LPO and H_2_O_2_ in control and test rats are illustrated in Figure 2a,b. When compared to Group 1, the levels of LPO and H_2_O_2_ were all markedly higher in the adipose tissue of the diabetic group. The values of stress markers in the adipose tissue of C. papaya medicament group were proficiently reduced compared to Group 2. Metformin medicament also showed a substantial decrease in these levels. These oxidative stress markers levels exhibited no changes in Group 5 rats.

### 3.3. Result of C. papaya on Enzymatic Antioxidants

**Figure 3:**
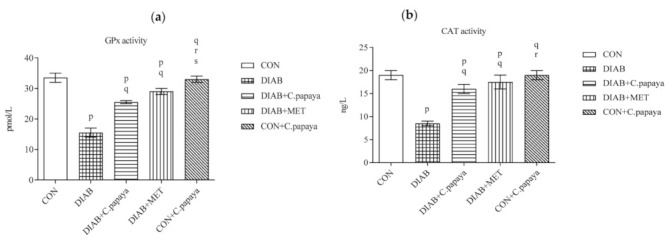

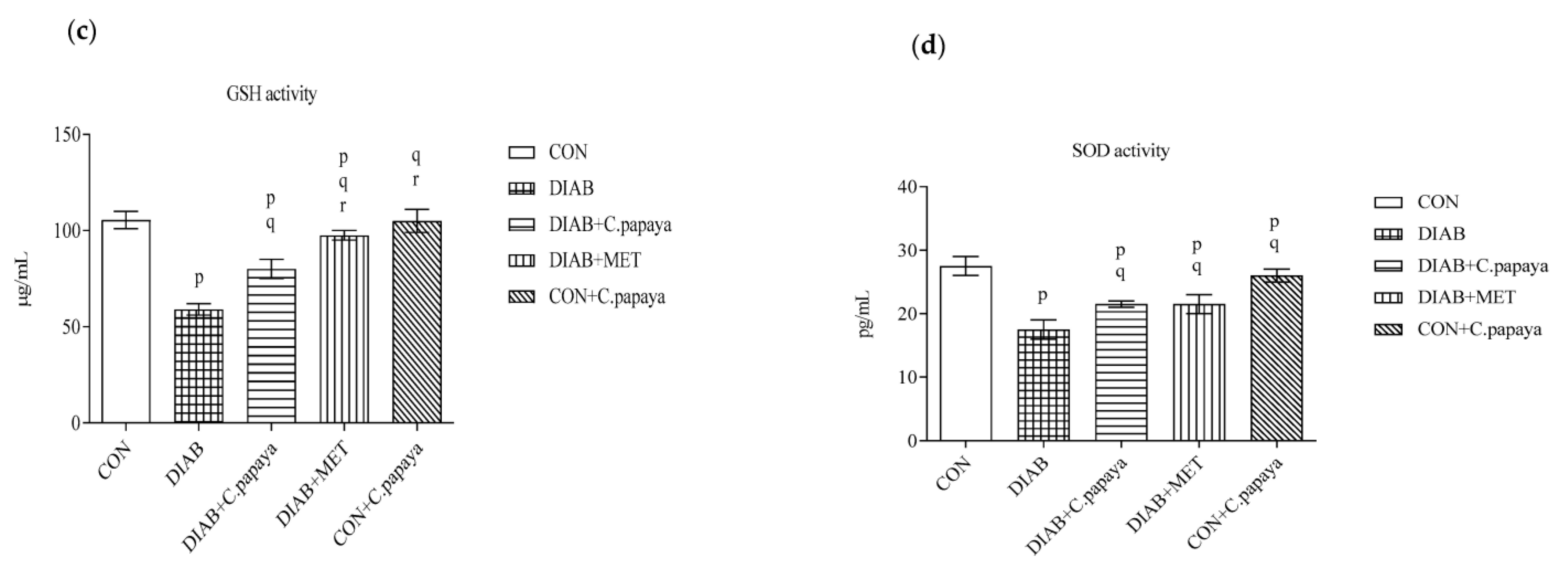
(**a**–**d**) Outcome of C. papaya on the enzymatic antioxidants levels in the control and treated rats. Each bar depicts the mean ± SEM of eight rats, with *p* < 0.05 designating statistically significant differences between the groups as follows: p—control; q—diabetes; r—T2DM rats + C. papaya; s—T2DM rats + Metformin.

The levels of catalase, glutathione peroxidase and superoxide dismutase in control and test rats are illustrated in Figure 3a–d. When compared to Group 1, the levels of the enzymes GPx, CAT, GSH and SOD were all markedly lower in the adipose tissue of the diabetic group. The values of antioxidant enzymes in the adipose tissue of C. papaya medicament group were proficiently augmented compared to Group 2. Metformin medicament also showed a substantial improvement in these levels. These enzymatic antioxidant levels exhibited no changes in Group 5 rats.

### 3.4. Effect of C. papaya on mRNA Expression of mTOR, TNF-α, IL-1β, IL-6 and IKKβ in Adipose Tissue

**Figure 4:**
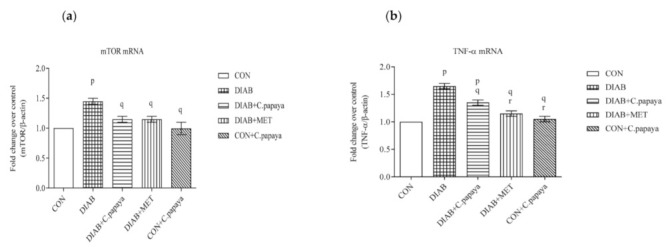

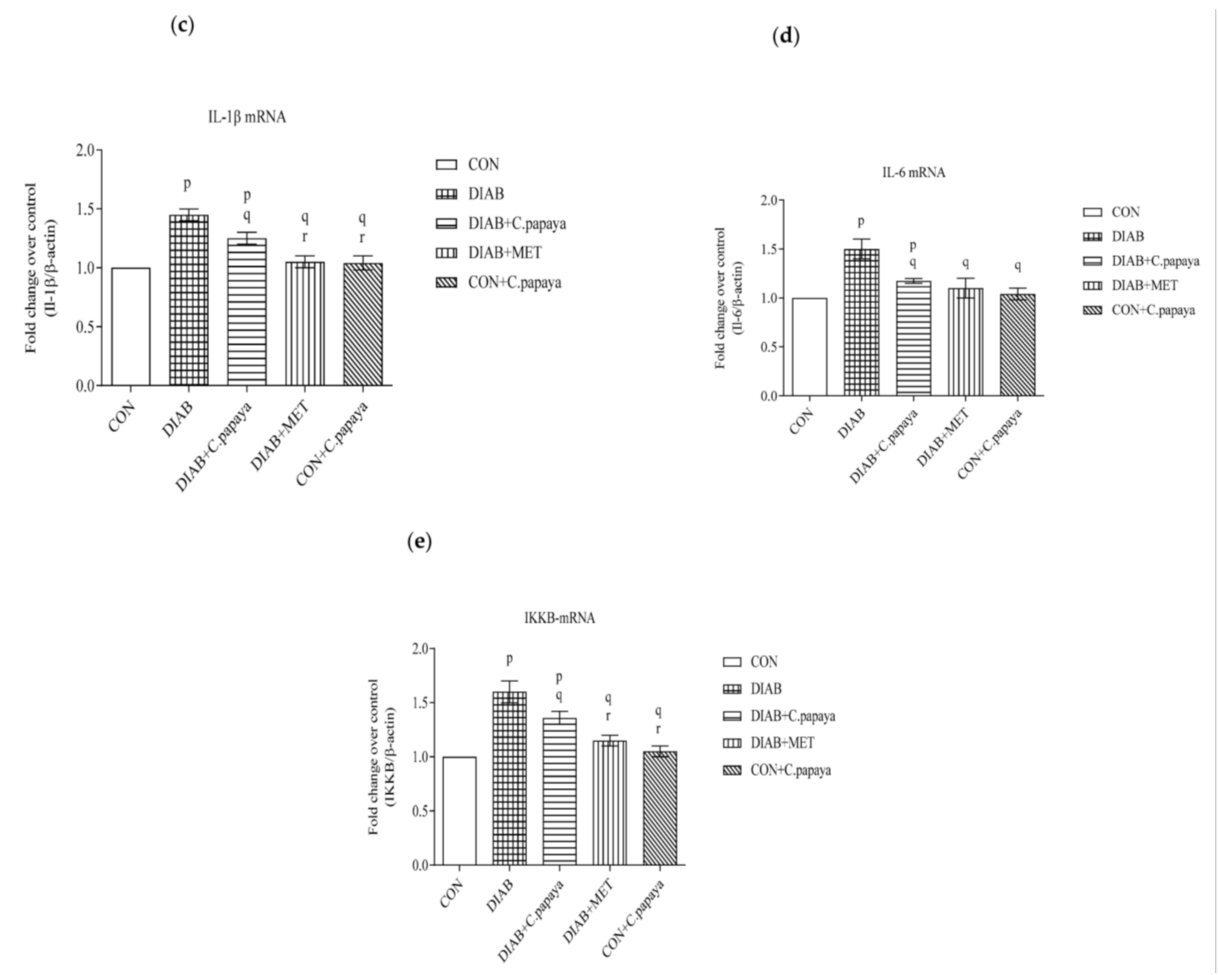
(**a**) Effect of C. papaya in control and treated T2DM rats, in terms of the levels of mTOR mRNA expression; (**b**) Effect of C. papaya in control and treated T2DM rats, in terms of the levels of IL-1β mRNA expression; (**c**) Effect of C. papaya in control and treated T2DM rats, in terms of the levels of IL-6 mRNA expression; (**d**) Effect of C. papaya in control and treated T2DM rats, in terms of the levels of TNF-α mRNA expression; (**e**) Effect of C. papaya in control and treated T2DM rats, in terms of the levels of IKKβ mRNA expression; Each bar depicts the mean ± SEM of eight rats, with *p* < 0.05 designating statistically significant differences between the groups as follows: p—control; q—diabetes; r—T2DM rats + C. papaya.

The impact of C. papaya on the mRNA expression of mTOR, TNF-α, IL-1β, IL-6 and IKKβ in the adipose tissue of all study groups are depicted in the Figure 4a–e. A significant surge in the gene expression levels of mTOR is observed in the T2DM rats, as shown in Figure 4a. The serine phosphorylation of IRS-1 by defective oxidative phosphorylation, inhibits mTORC1 and, in turn, insulin signaling [32]. In spite of that, C. papaya administration ameliorated the gene expression in the adipose tissue similar to the treatment of metformin. Pro-inflammatory cytokines, such as IL-1β, IL-6, and TNF-α, are produced in adipose tissues during pathogenesis of metabolic syndrome. Figure 4b–d displayed the elevated expression levels of IL-1β, IL-6, and TNF-α were elevated in the diabetic group 2 compared to group 1 control rats. The medicament of C. papaya brought down the levels of pro-inflammatory cytokines close to normalcy when compared to the treatment of standard drug, metformin. Over activation of IKKβ causes increased production of pro-inflammatory cytokines. Figure 4e shows substantial upsurge in the mRNA levels of IKKβ in the adipose tissue of diabetic group. The C. papaya medicament reduced the levels of IKKβ gene expression in the fatty tissue of diabetic rats.

### 3.5. Result of C. papaya on the Histopathological Changes in the Adipose Tissue

**Figure 5:**
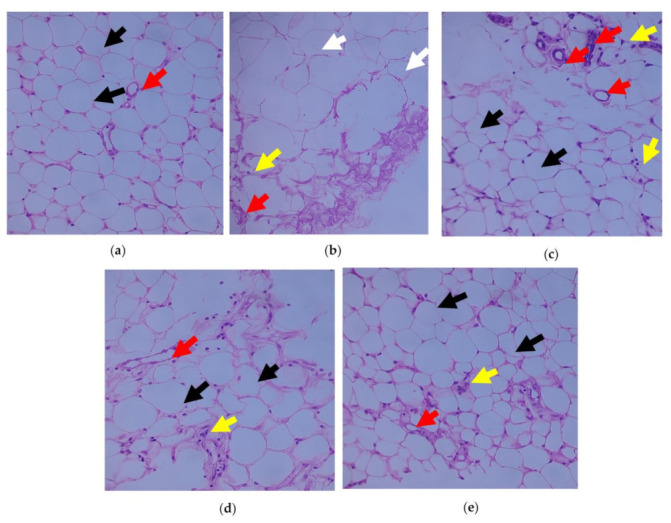
Outcome of C. papaya on the histopathology of adipose tissue: (**a**) Control rats; (**b**) T2DM rats displayed necrosed adipocytes with loss of nucleus (black arrow) when compared to the control; (**c**) T2DM rats + C. papaya showed large and small adipocytes when analyzed with T2DM rats; (**d**) T2DM rats + metformin mostly showed small adipocytes than large adipocytes when matched with the control rats; (**e**) control rats + C. papaya. Red arrow—blood vessels; yellow arrow—inflammatory cells.

The adipose tissue histological sections are shown in Figure 5a–e. When compared to control rats, Group 2 rats displayed necrosed adipocytes and increased fat storage and congested blood vessels due to a high-fat diet. Group 3 T2DM rats significantly reinstated the microstructure of the adipose tissue when compared to Group 4.

### 3.6. Outcome of C. papaya on the Immunohistochemical Changes in the Adipose Tissue

**Figure 6:**
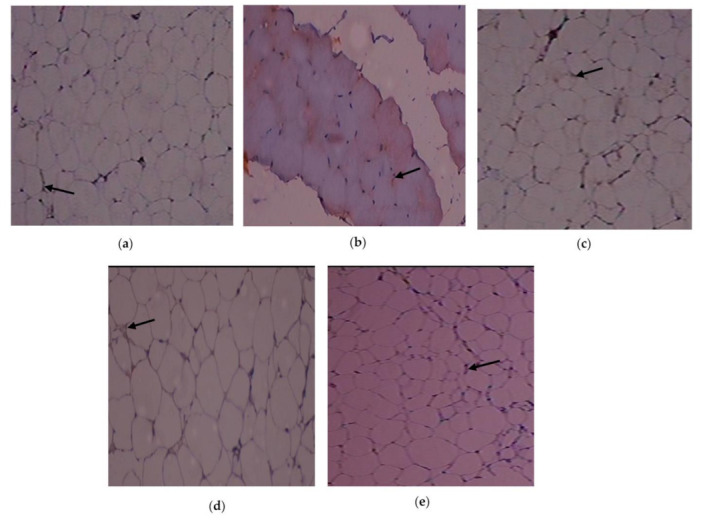
IL-1β protein levels as determined by an immunohistochemistry experiment (100×): (**a**) control rats; (**b**) T2DM rats; (**c**) T2DM rats + C. papaya; (**d**) T2DM + metformin; (**e**) control rats + C. papaya.

**Figure 7:**
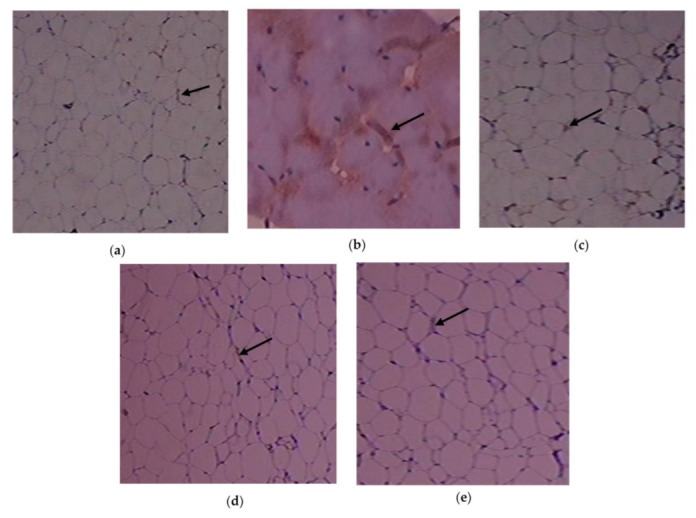
IL-6 protein levels as determined by an immunohistochemistry analysis (100×): (**a**) control rats; (**b**) T2DM rats; (**c**) T2DM rats + C. papaya; (**d**) T2DM rats + metformin; (**e**) control rats + C. papaya.

**Figure 8:**
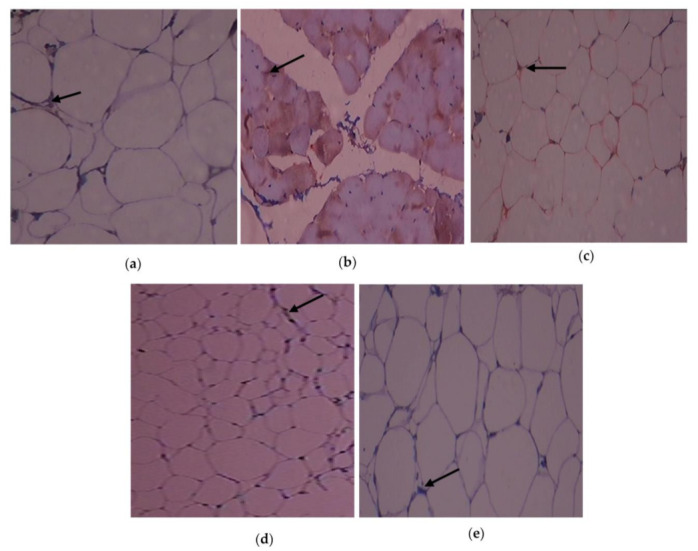
TNF-α protein levels as determined by an immunohistochemistry analysis (100×): (**a**) control rats; (**b**) T2DM rats; (**c**) T2DM rats + C. papaya; (**d**) T2DM rats + metformin; (**e**) control rats + C. papaya.

**Figure 9:**
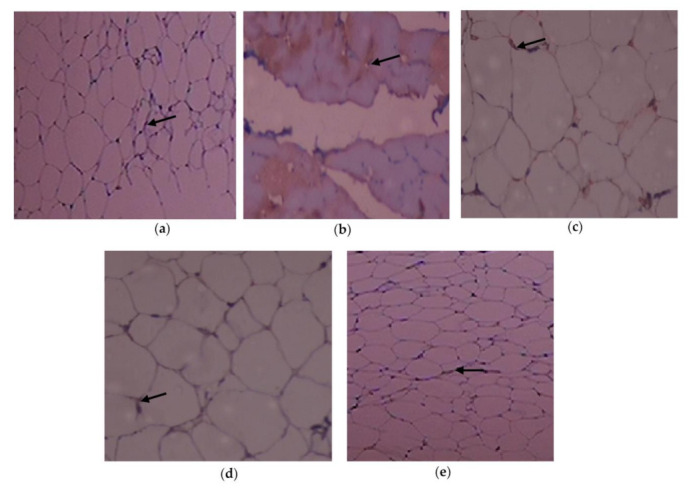
mTOR protein levels as determined by an immunohistochemistry analysis (100×): (**a**) control rats; (**b**) T2DM rats; (**c**) T2DM rats + C. papaya; (**d**) T2DM rats + metformin; (**e**) control rats + C. papaya.

**Figure 10:**
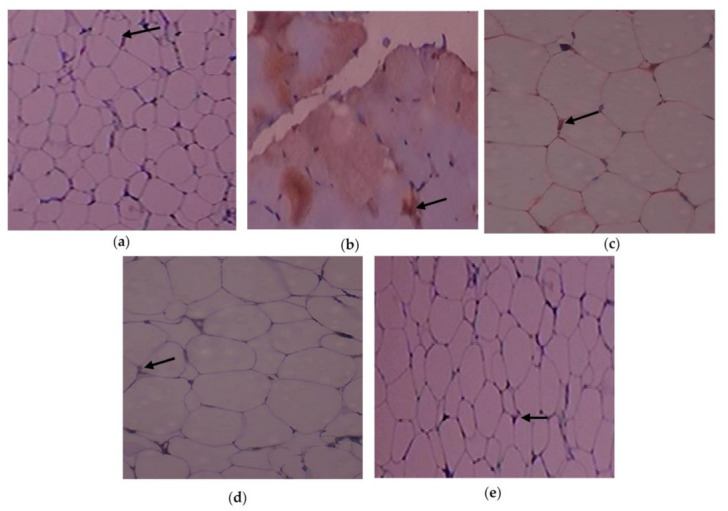
IKKβ protein levels as determined by an immunohistochemistry analysis (100×): (**a**) control rats; (**b**) T2DM rats; (**c**) T2DM rats + C. papaya; (**d**) T2DM rats + metformin; (**e**) control rats + C. papaya.

The immunohistochemistry alterations of IL-1β in the experimental and control rats are shown in Figure 6a–e. Animals in Group 2 had more IL-1β expressed in adipose tissue. As demonstrated in Group 3, C. papaya treatment brought down the IL-1β levels. In the T2DM adipose tissue after receiving metformin treatment, Group 4 rats similarly showed a significant reduction in IL-1β levels. Rats in Group 5 did not exhibit any notable alterations. The expression of the IL-6 protein in the five groups under study is shown in Figure 7a–e. Administration with C. papaya decreased the levels of IL-6 in the same adipose tissue, which was elevated in the diabetic group. Similarly, treatment with metformin showed a significant decrease in IL-6 levels. No discernible alterations in the protein levels were seen in the control rats given C. papaya treatment. Figure 8, Figure 9 and Figure 10a–e display the immunohistochemical changes of TNF-α, mTOR and IKKβ in the experimental and control rats. The T2DM group rats exhibited elevated levels of TNF-α, mTOR and IKKβ in the fatty tissue. The administration of C. papaya lowered these protein levels in the diabetic rats of Group 3, similar to that of metformin treated Group 4 rats. There were no noticeable alterations in the Group 5 rats.

### 3.7. Molecular Docking

#### 3.7.1. Molecular Docking Results of IL-1β, IL-6 and IKKβ

**Figure 11:**
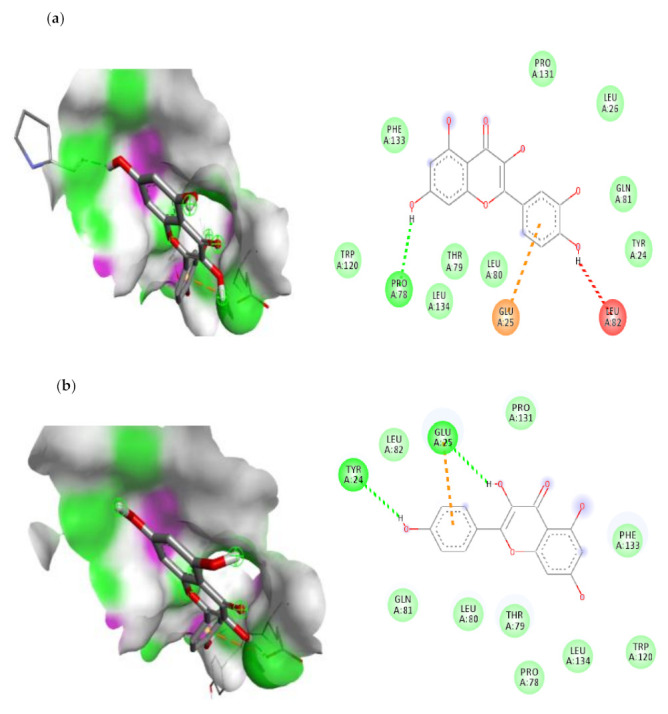
The top two compounds and its molecular interaction with IL-1 β; (**a**) quercetin; (**b**) kaempferol.

**Figure 12:**
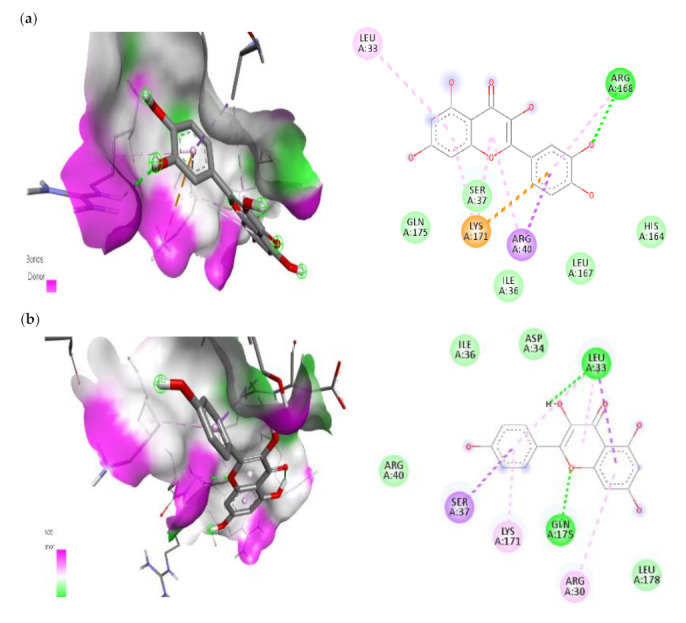
The top two compounds and its molecular interaction with IL-6; (**a**) quercetin; (**b**) kaempferol.

**Figure 13:**
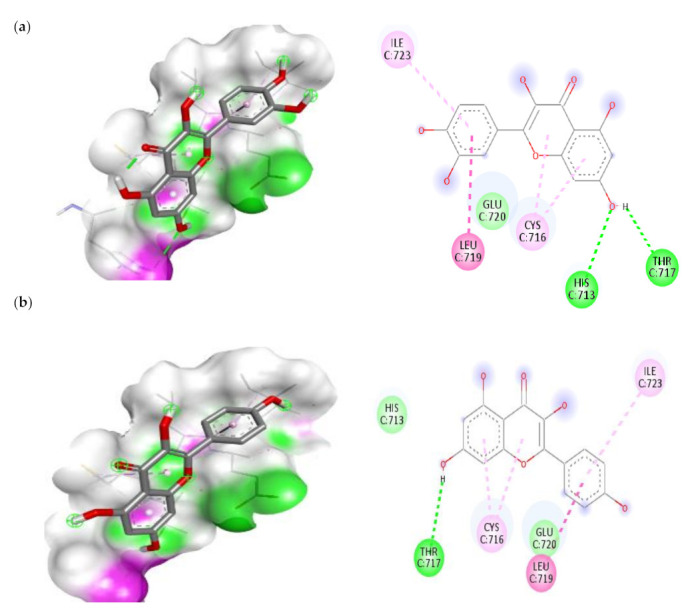
The top two compounds and its molecular interaction with IKKβ; (**a**) quercetin; (**b**) kaempferol.

Quercetin had the highest docking score on IL-1β followed by Kaempferol, Ferulic acid and Caffeic acid. The order of IL-6 binding affinity was as follows: Quercetin is superior to chlorogenic acid, kaempferol and rutin. The order of IKKβ binding affinity was as follows: Quercetin > Rutin > Kaempferol > Chlorogenic acid. According to Lipinski’s rule of five, the findings of ADME prediction were conducted in our prior work. Lipinski’s rule of five was not met by rutin or chlorogenic acid, hence we were unable to use these two compounds in our subsequent investigation. The visualization of the molecular docking complex was carried out through BIOVA discovery studio software and the interaction of IL-1β, IL-6 and IKKβ with best compounds were shown in Figure 11, Figure 12 and Figure 13. From these results, we observed that quercetin showed the best binding affinity with all the three target proteins (Table 3). With IL-1β protein quercetin form the one conventional bond interaction with PRO-78 and one Pi-Anion interaction with GLU-25 and also, it showed the strong binding with binding energy of −6.3 kcal/mol.

There are five types of bonds formed when a ligand attaches to a target protein: the normal hydrogen bond, the carbon hydrogen bond, the unfavourable donor-donor bond, the pi-sulfur bond and the pi-alkyl bond. Depending on the type of hydrogen bond, conventional hydrogen bonds and carbon hydrogen bonds play a function in binding ligand pockets. IL-6 protein forms four distinct interactions with quercetin. It forms a typical hydrogen bond with the IL-6 ARG-168 residues. With SER-37 and LEU-33, it also forms the two Pi-Alkyl interactions. With LYS-171 amino acid, quercetin forms a Pi-sigma connection. All of these interactions demonstrate quercetin’s ability to bind to the IL-6 protein. In the ligand-macromolecule interaction, hydrogen and/or hydrophobic bonds may also be formed. The bonds were critical intermolecular interactions for stabilising ligands in the conformational context of the macromolecule. In the current study, quercetin forms two hydrogen bonds with HIS-713 and THR-717, as well as three hydrophobic bonds with LEU-719, CYS-716, and ILE-723 to the IKKβ (Table 3). In this work, we discovered that quercetin and kaempferol had antidiabetic effects via suppression of IL-1β, IL-6, and IKKβ, suggesting that they may be promising candidate for the treatment of diabetes.

#### 3.7.2. Molecular Docking Results of TNF Alpha and mTOR

The docking analysis of seven compounds derived from C. papaya was performed against TNF Alpha and mTOR and then top two compounds were carefully chosen depending on their scoring and binding patterns. The compounds Caffeic acid and Trans-Ferulic acid showed the good binding with TNF Alpha and mTOR protein based on their scoring parameters.

**Figure 14:**
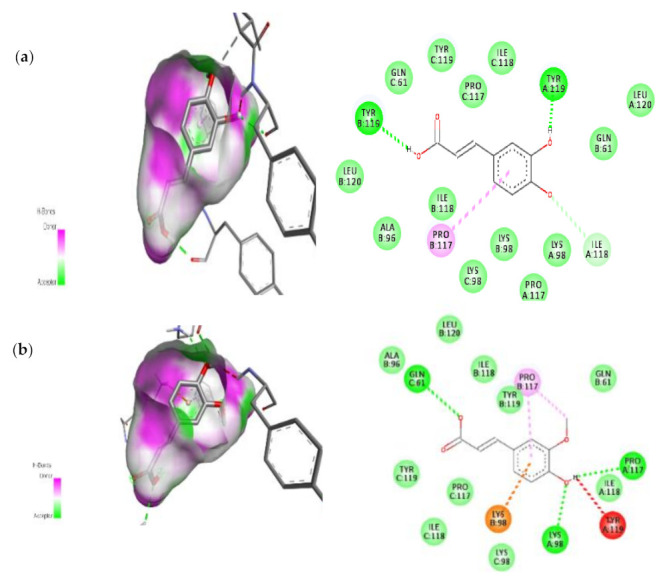
The top two compounds and its molecular interaction with TNF-α; (**a**) Caffeic acid; (**b**) Transferulic acid.

**Figure 15:**
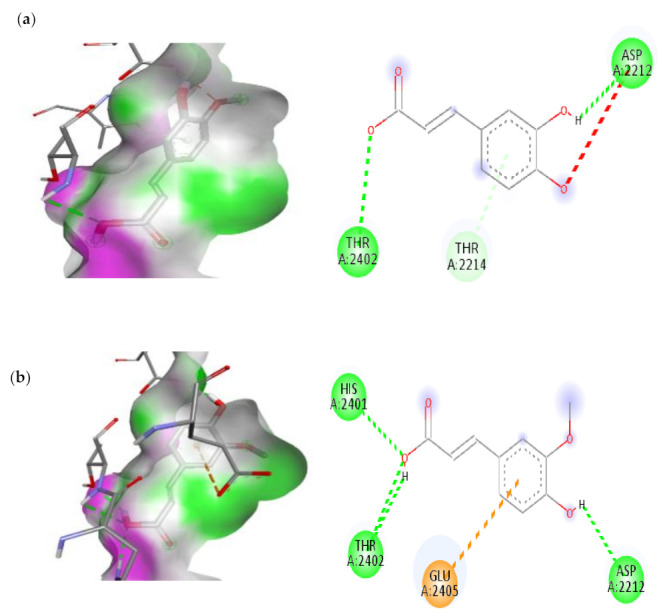
The top two compounds and its molecular interaction with mTOR; (**a**) Caffeic acid; (**b**) Transferulic acid.

Detailed interaction of these compounds with target proteins were shown in Figure 14 and Figure 15 and the scoring parameter was listed in Table 4. These amino acids have been modified as active amino acids of the catalytic cleft because they are frequently associated in the binding interaction of each compound with receptor protein. Many earlier works have revealed that C. papaya contains a variety of compounds that help to lower blood sugar levels, and these studies back up our findings.

### 3.8. Molecular Dynamics Simulation

#### 3.8.1. Trajectory Analysis of TNF-Alpha with Selected Compounds Complexes

Monitoring the stability profile of Caffeic acid and Trans-Ferulic acid with TNF-α by means of the GROMACS command line gmx rmsd to evaluate their relative RMSD values across simulated runs. RMSD often provides an indication of the magnitude of a group of atoms’ (protein, ligand or even ligand–protein complex) divergence from the corresponding initial reference structure [33]. Therefore, due to changes in the analyzed molecule’s shape, elevated RMSD values could be indicative of severe instability. Furthermore, ligands exhibiting large RMSD values for their specific ligand-protein complex would indicate inadequate ligand accommodation within the examined pocket during the course of the used MD simulation timeframes. Using the GROMACS programme, a 100-ns MD simulation was conducted to evaluate the model’s stability. Depending on the RMSD, RMSF and Rg values, as well as the H-bond-interaction, the stability of the molecules was analysed.

**Figure 16:**
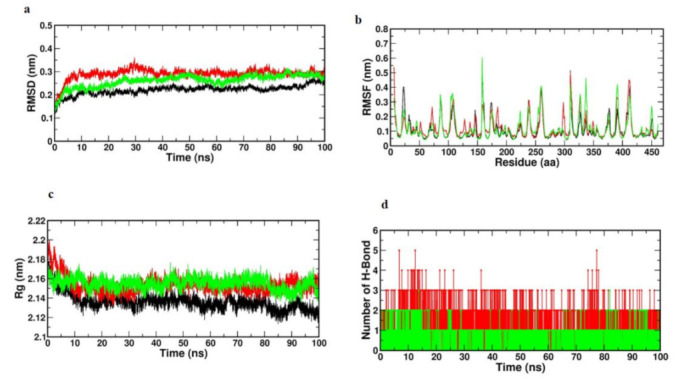
Binding stability analysis of the TNF-α with Caffeic acid and Trans-Ferulic Acid during 100 ns molecular dynamics simulation (Black—apo; Red—Caffeic acid; Green—Trans-Ferulic Acid); (**a**) Root Mean Square Deviation RMSD; (**b**) Root Mean Square Fluctuation RMSF; (**c**) Radius of gyration (RG); (**d**) H-bond interaction.

Figure 16a displays the RMSD values of each frame corresponding to time. This RMSD graph demonstrates that Caffeic acid and Trans-Ferulic acid exhibited stable motion throughout the MD simulation. Only Caffeic acid exhibited a slight variance between 10 and 30 ns, following which it also reached a stable condition. This indicates that the complicated molecules that were docked did not fluctuate significantly.

Based on the RMSF values obtained from the 100-ns MD simulation, the volatility of the individual amino acid residues can be explained. The highest and lowest RMSF values were 0.6 and 0.5 nm, correspondingly. The low average RMSF revealed that individual amino acid residues were stable in the protein’s dynamic state during the MD simulation. Figure 16b depicts a plot of RMSF values versus the number of amino acid residues. The graph demonstrates that amino acid residues at positions 100–150, 300–350 and 420 changed somewhat relative to the other places. During the MD simulation, the remaining amino acid residues were determined to be quite stable.

To get further insight into the examined complex’s stability, the GROMACS “gmx gyrate” command script was used to monitor the radii of gyration (Rg) along the whole MD trajectories. The entire stability of the ternary configuration of a ligand or protein is determined and Rg is the mass-weighted RMSD of a group of atoms with respect to their shared mass centre. Therefore, minimal Rg values that plateau around the average value would indicate sustained compactness of the tested molecule. Using Rg values, the stiffness of the protein system was then evaluated. Figure 16c’s Rg values were utilised to help the interpretation of the secondary structure. Figure 3c demonstrates that the Rg value increased at the initial point, indicating that the system’s stability was initially disturbed. After 10 ns, there was no fluctuation in the figure, indicating that the protein system remained stable.

Because it influences drug selectivity, metabolism, and adsorption, the hydrogen bond is critical for ligand binding to receptors. The potential number of hydrogen bonds in the complexes was therefore calculated during the 100 ns simulation period. In the compound, about two to three hydrogen bonds were found. This showed that the selected compounds had a considerable affinity for TNF-α (Figure 16d).

The binding energy was determined by summing the polar, non-polar and non-bonded interaction energies and the hit compounds demonstrated increased binding affinity. Depending on the MM-PBSA data, we noticed that Caffeic acid and Trans-Ferulic acid had an effective binding affinity for TNF-α in terms of binding energy (Table 5).

#### 3.8.2. Molecular Dynamic Simulation of mTOR

**Figure 17:**
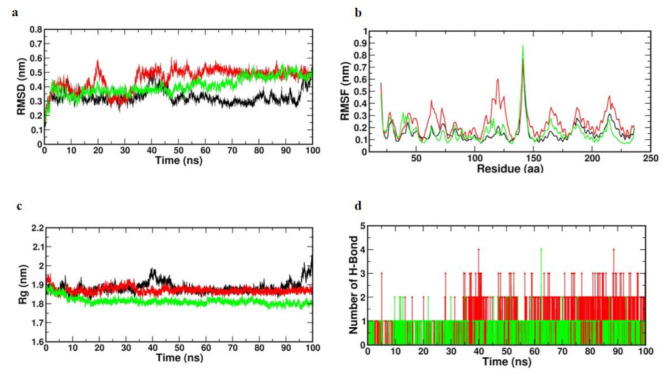
Binding stability analysis of the mTOR with Caffeic acid and Trans-Ferulic Acid during 100 ns molecular dynamics simulation (Black—apo; Red—Caffeic acid; Green—Trans-Ferulic Acid); (**a**) Root Mean Square Deviation RMSD; (**b**) Root Mean Square Fluctuation RMS; (**c**) Radius of gyration (RG); (**d**) H-bond interaction.

To analyse the variation of compounds across a 100 ns trajectory period, the Root Mean Square Deviation (RMSD) of all protein-containing complexes was determined. The RMSD plot of all protein–ligand complexes demonstrated their stability. In this investigation, Caffeic acid simulations of 20 and 35 ns showed some variance. After that, it exhibited stable mobility for the duration of the experiment. Consequently, the scrutiny of the RMSD plot demonstrated that protein and complexes attained acceptable compactness in 100 ns and generated a consistent trajectory. Figure 17a depicted the RMSD graph of both complexes.

Utilizing the Root Mean Square Fluctuation (RMSF) measurement at a given temperature and pressure, the local variations of chemicals and protein chain residues were analysed. Throughout the 100 ns trajectory period, only few alterations were observed in the protein–caffeic acid complex’s constituent residues. Figure 17b illustrates all complexes fluctuations. At the beginning, both complexes exhibited fluctuations, however the Caffeic acid complex exhibited fluctuations only at the 120th and 150th positions, after which it assumed a stable form.

By estimating the structural compactness along the MD trajectories, the Radius of gyration (Rg) study was performed to determine the stability of protein–ligand complexes. The computation of Rg was also influenced by whether the protein and complexes system were folded or unfolded. In this work, 100 ns trajectories were utilised for the determination of the Radius of gyration. Figure 17c depicts the Rg graph and the result demonstrated that the entire complexes had comparable and consistent Rg values and good stability.

Figure 17d depicts the overall number of hydrogen bonds generated throughout the 30-ns simulation period. Initially, both complexes exhibited a single hydrogen bond contact, however, after a period of time, they exhibited more than two hydrogen bond interactions. It was established that the movement of both complexes was stable during the simulation time. The binding free energy was calculated by MMGBSA, and its analysis of particular chemicals is detailed in Table 5.

### 3.9. Molecular Dynamics Simulation of IL-1Beta, IL-6 and IKKβ

#### 3.9.1. Root Mean Square Deviations RMSD

**Figure 18:**
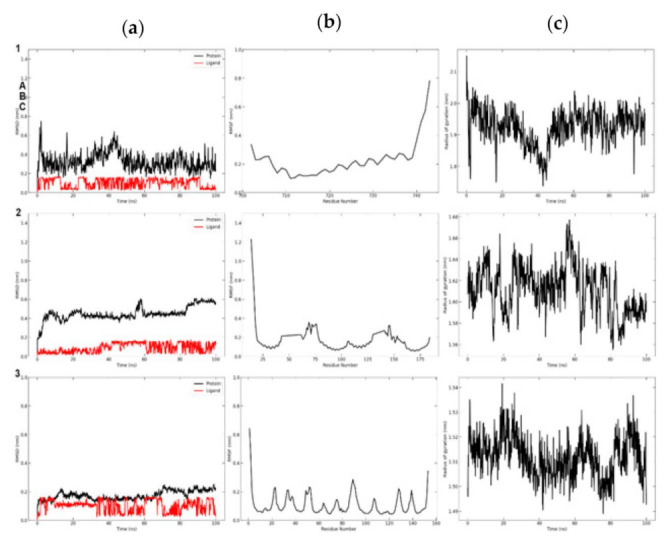
From left to right: (**a**) RMSD, (**b**) RMSF and (**c**) Radius of gyration of the complexes during 100 ns MD simulation. IKKβ (top), IL-6 (middle) and IL-1β (bottom); (apo—black; red—quercetin).

RMSD was calculated on the basis of ‘Backbone’ atoms using GROMACS program. RMSD graph (Figure 18, Column a) for protein complex shows that the structure remained constant during the simulation time with some fluctuation within the range of ~1 Å, which is a normal behavior of globular protein. Ligand RMSD was calculated for the ligand based on ligand’s atoms using GROMACS program and it is shown in (Figure 18, Column a).

#### 3.9.2. Root Mean Square Fluctuations RMSF

Using the GROMACS tool, RMSF for the protein complex was estimated based on “C-alpha” atoms. Overall, the fluctuation intensity remains below 2.5 Å except for some residues which represent a loop or turn in the protein (Figure 18, Column b).

#### 3.9.3. Radius of Gyration (Rg)

The radius of gyration was measured for the complex on the basis of ‘C-alpha’ atoms by means of GROMACS program. The minor variation within the 1 Å Rg value during the MD simulation time indicated a slight opening and closing of the N and C terminal domains (Figure 18, Column c).

#### 3.9.4. Hydrogen Bonds (Protein-Ligand)

**Figure 19:**
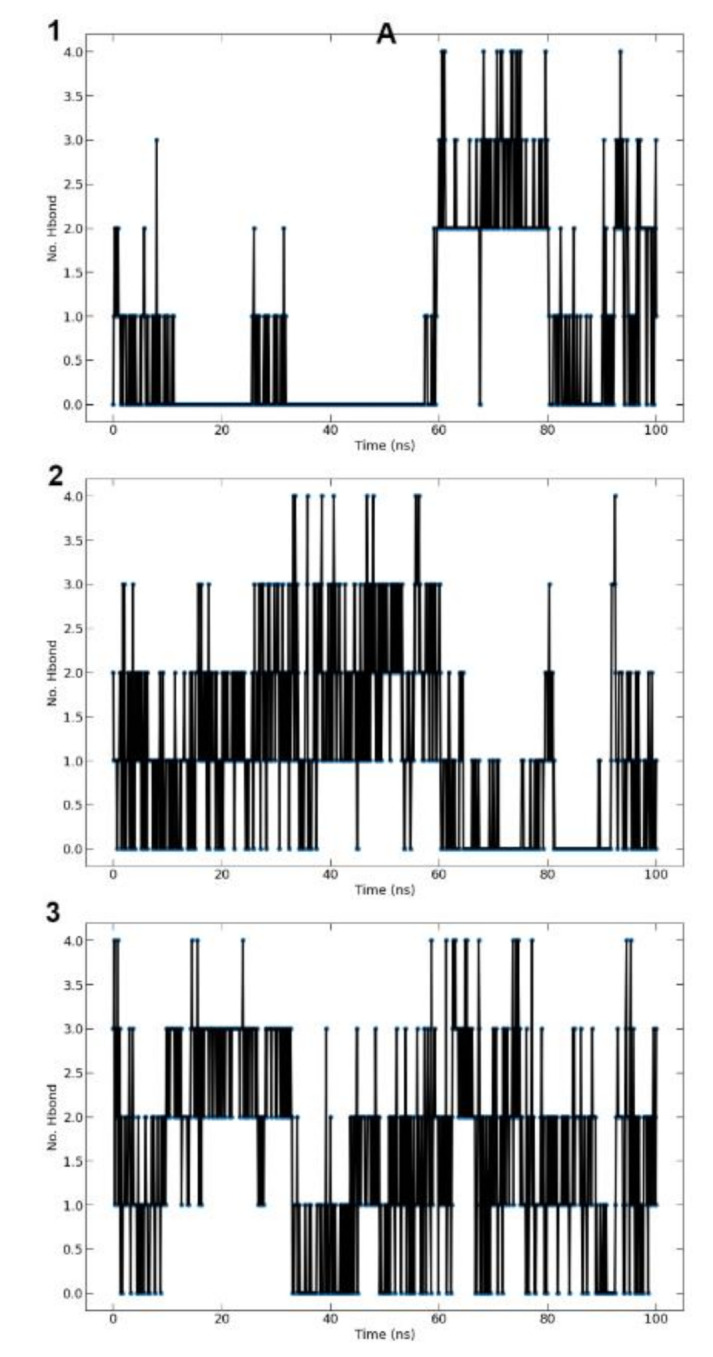
From left to right: (**A**) Hydrogen Bonds (Protein-ligand) for the complexes during 100 ns MD simulation. IKKβ (1), IL-6 (2) and IL-1β (3).

The over-all number of hydrogen bonds formed between ligand and protein throughout 100 ns of the simulation time are shown in (Figure 19). Quercetin showed stable hydrogen bonds with the selected target proteins.

#### 3.9.5. MMPBSA Binding Energy

The Molecular Mechanics/Poisson Boltzman Surface Area (MM/PBSA) approach was chosen for rescoring complexes because, when compared to other force field-based computational free energy methods, it computed the free energy of binding the quickest. The MM/PBSA calculation was performed using g-mmpbsa software. In accordance with MM-PBSA data, we noted that quercetin had a strong binding affinity against IL-1β, IL-6 and IKKβ. The estimated binding free energies are depicted in Table 6.

## 4. Discussion

Regarding acting as the body’s primary triglyceride storage organ, adipose tissue is considered to be an active endocrine organ. A variety of molecules known as adipokines, which are produced and secreted by adipose tissue, allow it to communicate with other central and peripheral organs. Some adipokines have levels that correlate with particular metabolic states and have the potential to have a direct impact on the system’s metabolic balance. A rising variety of pathological alterations in several organs, including obesity, type 2 diabetes, hypertension, cardiovascular disease, and others, have been linked to a dysregulation of adipokines [34,35]. The primary secretory molecules and important signaling pathways connected to insulin signaling are significantly altered in obese people, which leads to the development of insulin resistance, which is primarily caused by increased inflammatory processes in adipocytes [36]. Healthy individuals’ adipose tissue mostly secretes anti-inflammatory adipokines like that of adiponectin, IL-4, IL-10, IL-13 and transforming growth factor (TGF-β). These adipokines all play a role in mediating physiological processes. Conversely, the fatty tissue of obese individuals exhibits increased fat collection and subsequently secretes higher quantities of pro-inflammatory adipokines, such as leptin and resistin [37,38,39]. These molecules trigger a positive feedback loop that boosts the formation of proinflammatory cytokines including TNF-α, IL-6, and IL-1β by changing the IKKβ/NF-kB and c-Jun N-Terminal kinase (JNK) pathways in adipose tissue [40,41]. These proinflammatory adipokines recruit dendritic cells, T-lymphocytes and macrophages into adipocytes, which results in abnormal lipid metabolism. Elevated levels of free fatty acids in the bloodstream cause the group of invading cells to trigger inflammatory signaling [41]. Proinflammatory cytokines respond in a cycle that intensifies these pathogenic processes, which then promotes immune cell infiltration, cytokine generation and disruption of the insulin signaling pathways [41,42]. Adipose tissue malfunctions cause the liver and skeletal muscles to have poor glucose homeostasis that sequentially cause systemic insulin resistance and T2DM. In our work, the ethanolic extract of C. papaya was given to the investigational rats to overcome the obesity spurred inflammation and progress insulin sensitivity in the fatty tissues of high fat diet- streptozotocin-incited T2DM rats.

Adipocytokines, which are highly expressed in adipose tissue, help to maintain homeostasis by balancing energy and inflammation [43]. Fluctuations in the levels of adipocytokines are considered to be a sign of adipose tissue malfunction. Additionally, adipocytokines may offer vital information about the pathophysiological causes of type 2 diabetes mellitus [44]. Adipocyte-derived hormones, including adiponectin and leptin, increase insulin sensitivity, a vital step in the aetiology of type 2 diabetes [43]. In normal conditions, the levels of serum leptin and resistin remain lowered while the adiponectin remains elevated. These adipocytokines are serum markers that contribute a key part in the homeostasis of carbohydrate and lipid metabolism that in due course regulate the inflammation and insulin signaling. Any variation in these adipocytokines’ levels signifies the dysfunctional of adipose tissue and the consequent induction of processes leading to insulin resistance [45]. Leptin and resistin are responsible for enhancing insulin resistance through various means. The downregulation of hepatic glycogenesis by adiponectin enhances insulin sensitivity in the liver [46,47,48]. In our present study, the levels of serum leptin and resistin were significantly raised and adiponectin was lowered in the T2DM group when analyzed with the control rats. The fluctuation in these inflammatory markers causes insulin resistance, which in turn leads to type 2 diabetes [49,50]. The treatment of C. papaya brought down the levels of these adipocytokines close to normalcy, such as that of standard drug treated group. Likewise, Ghanbari, et al. [51] reported that aqueous and alcoholic extracts of Artemisia annua L. augmented the insulin resistance in high fat diet-streptozotocin induced T2DM mice with a surge in adiponectin and diminution in leptin and resistin production in fat cells. Ansari et al. and his co-workers reported in their work the effect of synthesized gold nanoparticles from Smilax glabra that modulated the levels of serum leptin and adiponectin in high fat diet-streptozotocin induced rat models [52]. Our study portrayed the hypolipidemic and antidiabetic properties of C. papaya and reduced the insulin resistance. The elevated levels of leptin promoted the generation of pro-inflammatory cytokines to generate reactive oxygen species and oxidative stress via monocyte or macrophage activation [53]. These proinflammatory cytokines dramatically increased the high expression of resistin in monocytes and macrophages, again leading to increased oxidative stress [54].

T2DM-incited dyslipidemia and oxidative stress increased ROS generation, which causes lipid peroxidation and membrane damage [55]. In our study, the elevated levels of LPO and H_2_O_2_ were viewed in T2DM group, causing an increase in the ROS formation. On the other hand, C. papaya significantly lowered LPO and H_2_O_2_ levels, which could be due to its antioxidant property. A similar work was reported by Othman et al. [56] as flavonoids from Ocimum basilicum extract decreased the oxidative stress markers in HFD-STZ diabetic rats.

Enzymatic antioxidants, such as SOD, GPx, CAT etc., get rid of the free radicals from cells and eliminate the oxidative stress. Reduced levels of these antioxidant enzymes induce insulin resistance that paves way to diabetes mellitus. In this present study, the enzymatic antioxidants CAT, SOD, GPx and GSH were appreciably reduced in the T2DM rats when analyzed with the control group. Medicament of C. papaya substantially improved antioxidant enzyme levels, considerably dropped ROS and dodged lipid peroxidation in the adipose tissue of T2DM animals as convincing as metformin. These results were rather like that of a study of Sadek [57], as C. papaya upregulated SOD, CAT, GSH, GPx levels and mitigated lipid peroxidation against acrylamide induced oxidative stress. A similar work was reported by Nain, et al. [58] and showed the anti-diabetic and antioxidant properties of Emblica officinalis Gaertn. leaves that could lower the oxidative stress in streptozotocin induced T2DM rats by activating the anti-oxidant enzymes. Our work demonstrated the treatment of C. papaya can result in the enhancement of the antioxidants levels like CAT, SOD, GSH and GPx that could protect the adipose tissues in the high fat diet-streptozotocin-induced diabetic rats against oxidative damage caused by the ROS.

**Figure 20:**
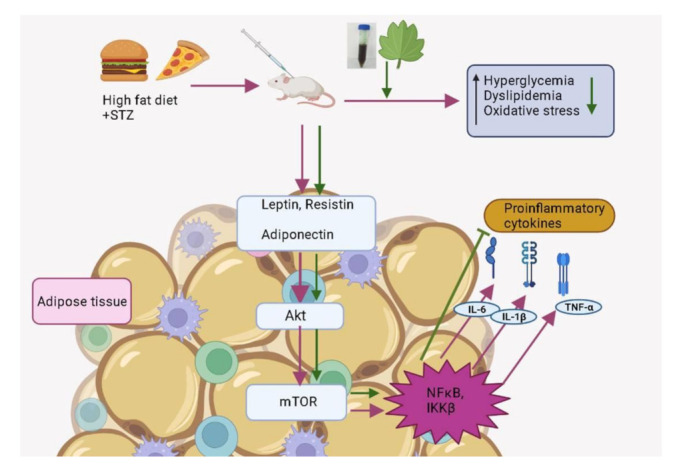
Summarising the effect of *C. papaya* on IL-1β/IL-6/TNF-α signalling molecules in high fat diet and streptozotocin induced type 2 diabetic rats. Pink arrow indicates the high fat diet induced development of inflammation while green arrow represents the anti-inflammatory potential by C. papaya through the downregulation of IL-1β/IL-6/TNF-α signaling molecules.

We assessed the gene expression of proinflammatory cytokines, such as IL-1β, IL-6 and TNF-α, as well as mTOR and IKKβ in the adipose tissues of the control and experimental rats in order to assess the ability of C. papaya to mitigate HFD-streptozotocin-incited inflammation and insulin resistance. Gouranton and his co-workers demonstrated in their study that lycopene had a moderating effect on the formation of IL-1β, IL-6 and TNF-α in adipose tissue and eventually might prevent or reduce the incidence of obesity-related diseases including insulin resistance [59]. Similarly, the fruit extract of Rubus idaeus L. dramatically reduced inflammation in hypertrophied adipocytes by suppressing the production and secretion of pro-inflammatory mediators (IL-6, TNF-α, IL-1β and leptin) and enhanced adiponectin and IL-10 expression and secretion [60]. In our study, Group 2 diabetic rats had an elevated mRNA expression of IL-1β, IL-6 and TNF-α, depicting increased inflammation vis-a-vis control Group 1. The treatment with C. papaya lowered the mRNA gene expression of IL-1β, IL-6 and TNF-α as close to that of metformin treatment. This exhibited the potentiality of C. papaya of suppressing the formation and secretion of pro-inflammatory cytokines. Apart from this, the C. papaya administration even brought down the mRNA levels of IKKβ and mTOR of gene expression when compared to the Group 2 diabetic rats (Figure 20). Likewise, Shokouh et al. [61] displayed, in a T2DM hepatic rat model, the expression of the mTOR gene was downregulated in the coffee groups to increase insulin sensitivity. Increased fat and elevated circulating amino acids in particular have been found to contribute to nutrient overload, which in turn increases mTOR activation which can result in insulin resistance in peripheral insulin-responsive tissues [62]. Ren et al. reported that the foxtail millet intake dramatically decreased the phosphorylation levels of IKKB and NF-κB in the hepatocytes of diabetic rats and could significantly reduce inflammation by suppressing the activation of the NF-κB signaling pathway [63]. In our work, the gene expression of mTOR and NF-κB were reduced by the intervention of C. papaya when matched to the HFD-streptozotocin incited diabetic rats. These clearly proved the anti-inflammatory potential of C. papaya to regulate the insulin sensitivity in diabetes mellitus.

Immunohistochemical investigations reinforced the antidiabetic and anti-inflammatory potential of C. papaya in the adipose tissue of investigational rats and displayed its impact on the IL-1β, IL-6, TNF-α, mTOR and IKKβ. In this present study, the outcome of the staining of these molecules was very much increased in diabetic fatty tissue when matched to the normal rats. The administration of C. papaya improved insulin sensitivity in adipose tissue and downregulated these protein targets implicated in the inflammatory signaling cascade to a level comparable to metformin. Shabani et al. [64] reported IL-1β, IL-6 and TNF-α were expressed less when resveratrol was administered. A similar study was displayed by Moruzzi et al. [65] that tart cherry juice and seeds diminished the excess levels of the pro-inflammatory cytokines. IKKβ protein levels were seen to be lowered in mice prostate by the treatment of apigenin as reported by Shukla et al. [66]. The efficacy of C. papaya ethanolic extract in controlling serum levels of leptin, adiponectin, resistin and enzymatic antioxidants, along with diminished levels of mRNA gene expression of IL-1β, IL-6, TNF-α, and IKKβ, would likely be explained by the immunohistochemistry staining in our investigation.

High fat diet-streptozotocin resulted marked enlargement of adipocytes and fat deposition in T2DM rats when analyzed with the control group. Remarkably, supplementation of C. papaya considerably reduced the size of adipocytes, on par with metformin. These results were similar with those of Mopuri et al. [67]. These changes brought about by C. papaya medicament might be due to the enhancement of enzymatic antioxidants, declination of pro-inflammatory cytokines and restoration of protein levels in the peripheral insulin targets.

Many antidiabetic drugs are currently overused, but their negative consequences make them unsuitable and dangerous to use. This dire situation necessitates the development of antihyperglycemic drugs with minimal aftereffects and maximum efficacy, so we investigated natural peptides with high affinity for glucose-regulating receptors in the current study. The anti-inflammatory effect and reinstatement of the protein levels in the proposed targets in this present study is due to the action of the bioactive compounds of C. papaya. The in-silico analysis done in this current work revealed the compounds of C. papaya that showed high binding affinity, molecular properties of protein and their drug-target interactions. Quercetin and caffeic acid showed the maximum interaction and these might have impacted on the protein targets of the inflammatory pathway in reinstating the altered effects in the adipose tissue against the development of pro-inflammatory cytokines. In silico, drug discovery is expected to make drug discovery faster, cheaper and more effective. Despite these advantages, computational biology techniques have had several drawbacks, such as the fact that various tools yield different answers for the same analysis, making it impossible to fully trust the results without additional research and lab validation.

## 5. Conclusions

According to the current research, C. papaya exhibited anti-inflammatory and anti-hyperglycemic activity by modulating inflammatory molecules such IL-1β, IL-6, TNF-α, IKKβ, and mTOR while restoring normal levels of antioxidant enzymes, oxidative stress markers and gene expression analysis in high fat diet fed type-2 diabetes experimental rats. In addition to these, we have also proposed via in-silico study, that caffeic acid and quercetin of C. papaya docked well with IL-1β, IL-6, TNF-α, mTOR and IKKβ. The current work shows, for the first time, that C. papaya prevents obesity-induced insulin resistance by reducing inflammatory events in the adipose tissues of type 2 diabetic rats fed an HFD diet and by controlling the IKKβ. Therefore, C. papaya holds immense promise for investigation in clinical studies aimed at finding effective and safe medications for the treatment of type 2 diabetes.

## Figures and Tables

**Table 1:** List of nucleotide sequences used.

S1. No	Name of Gene	Gene Specific Primers	Reference
1	Beta actin	Sense primer: 5′-CGC GAG TAC AAC CTT CTT GC-3′ Anti-sense primer: 5′-CGT CAT CCA TGG CGA ACT GG-3′	[23]
2	mTOR	Sense primer: 5′-TTG AGG TTG CTA TGA CCA GAG AGA A-3′ Anti-sense primer: 5′-TTA CCA GAA AGG ACA CCA GCC AAT G-3′	[24]
3	TNF-α	Sense primer: 5′-ACT GAA CTT CGG GGT GAT TG-3′ Anti-sense primer: 5′-GCT TGG TGG TTT GCT ACG AC-3′	[25]
4	IL-1β	Sense primer: 5′-TAC CTA TGT CTG GCC CGT GGA G-3′ Anti-sense primer: 5′-ATC ATC CCA CGA GTC ACA CAG G-3′	[26]
5	IL-6	Sense primer: 5′-TTC TCT CCG CAA GAG ACT TCC-3′ Anti-sense primer: 5′-TTC TGA CAG TGC ATC ATC GCT-3′	[23]
6	IKKβ	Sense primer: 5′-GCA CCC TGG CCT TTG AAT G-3′ Anti-sense primer: 5′-TCC GTT CAA GTC CTC GCT AAC A-3′	[27]

**Table 2:** List of C. papaya compounds.

Sl. No	Compound Name
i.	Transferulic acid
ii.	Quercetin
iii.	Rutin
iv.	Chlorogenic acid
v.	Kaempferol
vi.	Protocatechuic acid
vii.	Caffeic acid
viii.	p-coumaric acid

**Table 3:** Molecular interactions and outcomes of the top two C. papaya compounds with chosen target proteins.

Sl. No	Compound Name	Binding Energy kcal/mol	Interacting Residues
		IL-1β	
1.	Quercetin	−6.3	PRO-78 (H-bond)GLU-25(Pi-Anion)
2.	Kaempferol	−6.3	TYR-24(H-bond) GLU-25(H-bond)
		IL-6	
1.	Quercetin	−6.1	ARG-168(H-bond) ARG-40(Pi-Sigma) SER-37(Pi-Alkyl) LYS-171(Pi-Cation) LEU-33(Pi-Alkyl)
2.	Kaempferol	−5.7	LEU-33(H-bond) GLN-175(H-bond) SER-37(Pi-Sigma) ARG-30(Pi-Alkyl) LYS-171(Pi-Alkyl)
		IKKβ	
1.	Quercetin	−5	HIS-713(H-bond) THR-717(H-bond) LEU-719(Pi-Amide) CYS-716(Pi-Alkyl) ILE-723(Pi-Alkyl)
2.	Kaempferol	−5	THR-717(H-bond) LEU-719(Pi-Amide)

**Table 4:** Evaluation of binding affinity based on propounded compounds and docked complexes.

Sl No.	Compound Name	Binding Energy kcal/mol	Interacting Residues
TNF-α			
1.	Caffeic acid	−6.7	TYR-119(H-Bond) PRO-117(Pi-alkyl)
2.	Transferulic acid	−6.6	GLN-61(H-Bond) LYS-98((Pi-alkyl)) TYR-119(H-Bond) LYS-98(Pi-cation)
mTOR			
1.	Caffeic acid	−6.5	ASP-2212(H-Bond) THR-2402(H-Bond)
2.	Transferulic acid	−6.4	ASP-2212(H-Bond) HIS-2401(H-Bond) THR-2402(H-Bond) GLU-2405(Pi-Anion)

**Table 5:** MM/PBSA estimation of top two compounds and chosen receptors’ docked complexes.

Compounds Name	VdW Energy (kJ mol^−1^)	Electrostatic Energy (kJ mol^−1^)	Polar Solvation Energy (kJ mol^−1^)	SASA Energy (kJ mol^−1^)	∆G
TNF-α					
Caffeic acid	127.312 ± 6.976	−3.326 ± 2.756	57.699 ± 8.530	−12.101 ± 0.693	−85.040 ± 9.971
Trans-Ferulic Acid	−140.334 ± 8.549	−2.877 ± 2.564	90.000 ± 16.505	−13.070 ± 0.740	−66.281 ± 16.686
mTOR					
Caffeic acid	−133.129 ± 8.888	−4.746 ± 2.692	49.135 ± 8.077	−11.507 ± 0.635	−100.248 ± 9.760
Trans-Ferulic Acid	−135.313 ± 9.188	−5.112 ± 4.418	59.421 ± 22.356	−12.723 ± 0.635	−93.726 ± 24.063

**Table 6:** Calculated binding free energies of the top compound-Quercetin [kJ/mol].

Complex	ΔG	Van Der Waal Energy	Electrostatic Energy	Polar Solvation Energy	SASA Energy
3BRV	−70.444 ± 38.771	−47.325 ± 30.022	−60.150 ± 68.143	43.763 ± 40.074	−6.732 ± 3.409
4NI9	−66.310 ± 42.238	−74.532 ± 31.307	−67.017 ± 70.124	84.696 ± 49.905	−9.457 ± 3.103
9ILB	−78.544 ± 25.532	−71.504 ± 25.995	−96.621 ± 47.023	98.843 ± 48.350	−9.263 ± 3.531

## Data Availability

The data presented in this study are available in this article.
